# A Case of COVID-19-Induced Vestibular Neuritis

**DOI:** 10.7759/cureus.8918

**Published:** 2020-06-30

**Authors:** Srikrishna V Malayala, Ambreen Raza

**Affiliations:** 1 Internal Medicine, Bayhealth Hospital, Dover, USA

**Keywords:** covid-19 disease, central vertigo, vestibular evaluations, novel covid-19

## Abstract

The World Health Organization (WHO) declared COVID-19, a novel coronavirus infection, as a pandemic in March 2020. Since the origin of the disease in Wuhan, China, understanding the pathophysiology, clinical presentation, screening guidelines, and management of the disease has been ever-evolving. Though respiratory pathologies have been the major complications of a COVID-19 infection, other presentations like abdominal pain, deep venous thrombosis, cardiomyopathy, and even acute cerebrovascular ischemic attacks have been reported.

We present a case of a young patient presenting with vertigo, possibly from COVID-19-induced acute vestibular neuritis. This is a 20-year-old Hispanic female patient presenting with intractable vertigo, nausea, and vomiting but without any typical symptoms like fever, cough, or shortness of breath. Initial examination and imaging ruled out an acute stroke. There was minimal improvement in her vestibular symptoms with the recommended COVID-19 treatment as of March 2020 (hydroxychloroquine and azithromycin) and symptomatic management. Her inflammatory markers were surprisingly normal all through the hospital course. She was then treated with oral prednisone and subsequently discharged home after a prolonged course of eight days.

The pathophysiology of COVID-19-induced vestibular neuritis could be similar to any other viral infection. Clinicians should consider COVID-19 in the differential diagnosis for patients presenting with similar symptoms, especially in areas of a high prevalence of this disease. Early diagnosis of COVID-19 in such cases is important for proper isolation, to minimize exposure and avoid further unnecessary investigations. These symptoms will just resolve with symptomatic management like any other case of vestibular neuritis without any further management that is specific for a COVID-19 infection.

## Introduction

In December 2019, a novel type of coronavirus otherwise designated as COVID-19 by the World Health Organization (WHO), was identified as the cause of a cluster of pneumonias in the city of Wuhan, Hubei Province of China [1]. The virus rapidly spread, resulting in an epidemic throughout China, eventually leading to a pandemic causing 490,000 worldwide deaths as of June 2020. [2]. Since then, the understanding of COVID-19 pathophysiology and clinical presentation has been ever-evolving.

Pneumonia appears to be the most frequent and the most serious manifestation of a COVID-19 infection, presenting as fever, cough, dyspnea, and bilateral infiltrates on chest imaging [3].

However, there are no specific clinical features that can as yet reliably distinguish COVID-19 from other viral respiratory infections and there have been many other presentations of COVID-19, including conjunctivitis, upper respiratory symptoms, cardiomyopathy, deep venous thrombosis, and even ischemic stroke.

There have been a few reports for COVID-19-induced or associated stroke, delirium, epileptic, and non-epileptic seizures, and non-specific neurologic syndromes presenting like encephalitis [4]. It is not clear whether these neurologic presentations are a direct result of the nervous system infection by the virus or through an indirect or inflammatory response to the cytokine storm.

We present a case of intractable nausea and vertigo in a patient with a COVID-19 infection, possibly a manifestation of acute vestibular neuritis from COVID-19. The patient’s identification has been kept confidential in the manuscript.

## Case presentation

A 29-year-old Hispanic female presented to the emergency room in the first week of April 2020 with sudden onset of severe vertigo, nausea, and vomiting two days prior to arrival. The only other symptom was generalized fatigue without any focal weakness in the extremities, fever, chills, and cough and phlegm or chest pain. She denied any diarrhea, abdominal pain, anosmia, or dysgeusia. She denied any accompanying symptoms such as tinnitus, hearing loss, feeling of unsteadiness, veering to one side when walking. She denied any prior history or prior episodes of vertigo, recent upper respiratory symptoms, or recent trauma. She complained of vertigo at rest and it worsened with any type of movement. She described it as "persistent" vertigo in every position, though she was not able to explain the direction in which movement made it worse.

She was working at a chicken plant in the local rural community, which had a huge cluster of COVID-19 infections.

On arrival at the emergency room, she had stable hemodynamics, was afebrile, had a blood pressure of 139/72 mmHg, and was saturating 100% on room air. She looked in severe distress from nausea, non-bilious vomiting, and severe vertigo. Neurologically, her cognition was intact, and the cranial nerve exam did not show defects. Fine finger movement and rapid alternating movements of the extremities were intact as well. It was difficult to identify nystagmus in her initial presentation due to the acuity of illness from the other symptoms. Further positioning maneuvers like Dix Hallpike could not be attempted for the same reason. Gait examination was deferred for the same reason.

**Figure 1:**
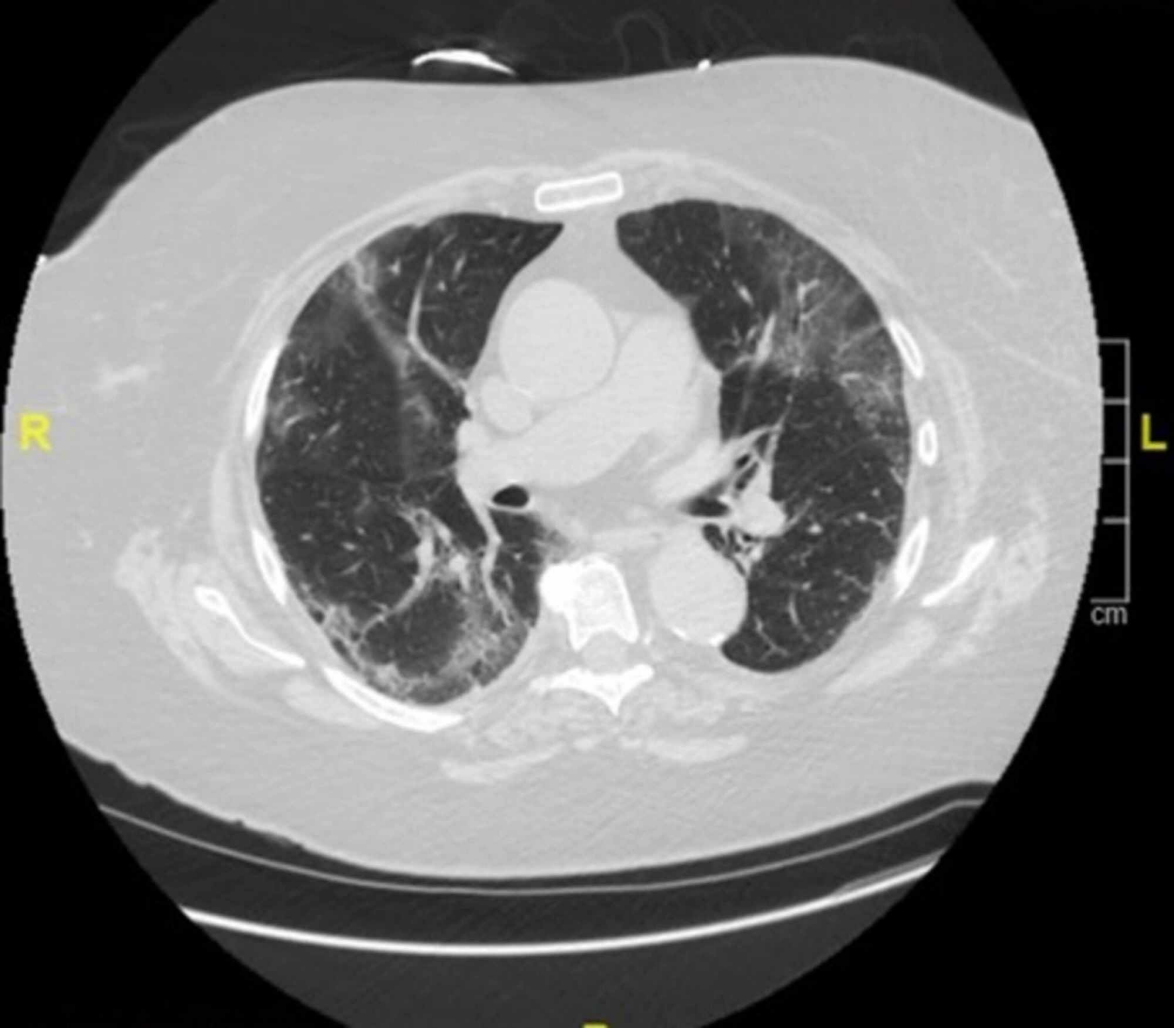
CT scan showing ground-glass capacities in the lung peripheries CT scan of the patient at the time of admission shows bilateral, ill-defined, ground-glass infiltrates.

Her urine toxicology was negative while white cell and platelet counts were within normal limits. Computed tomography (CT) scan of the head without contrast showed no acute pathology. With the presentation of nausea, abdominal pain, and non-bilious vomiting, a CT chest/abdomen and pelvis without contrast was done in the emergency room, which showed multifocal, bilateral, peripheral, ill-defined, rounded, ground-glass opacifications, features that could be consistent with acute COVID-19 pneumonitis (Figure 1).

Acute thromboembolic stroke was a differential that had to be ruled out at the time of admission, and MRI/MRA of the brain was checked. They did not show any acute findings.

Acute vestibular neuritis was the initial admission diagnosis and she was managed symptomatically with anti-emetics, meclizine, and benzodiazepines as needed.

In the emergency room itself, the COVID-19 polymerase chain reaction (PCR) test was requested due to the CT chest findings. Upon testing positive, she was started on hydroxychloroquine and azithromycin, which at the time was thought to be a promising treatment for COVID-19 infection [5]. Interestingly, she never had a fever or any respiration symptom all through her hospital course. All the inflammatory markers, including erythrocyte sedimentation rate (ESR), C-reactive protein (CRP), and ferritin, were within normal limits and so were the coagulation parameters, including d-dimer, fibrinogen, and platelet count.

Her symptoms were refractory to the then considered "traditional" management of a COVID-19 infection, and she was subsequently treated with intravenous steroids along with supportive care. She also received vestibular rehabilitation from physical and occupational therapy for a brief period.

Her symptoms gradually improved, and she was able to tolerate a diet and eventually discharged home after an eight-day stay in the hospital. The symptoms were quite refractory to the above-mentioned treatment and it took more than a week for her to recover and become asymptomatic. After discharge, she was advised to quarantine herself for seven more days.

## Discussion

There is a severe dearth of literature on COVID-19 infection as of spring and summer 2020. It is a reasonably established fact that COVID-19 may predispose to both venous and arterial thromboembolic disease due to excessive inflammation, hypoxia, immobilization, and diffuse intravascular coagulation (DIC) [6].

The neurologic manifestations of COVID-19 were reported in a few cases presenting as non-specific symptoms like dizziness, headache, and impaired consciousness [4-7]. There are reports of more acute and severe acute cerebrovascular presentations like ataxia and seizures [4-7]. Even cranial nerve and peripheral nervous system symptoms, including impaired taste, smell, vision, and nerve pain, have been reported [4-7]. In a case series published in the Journal of American Medical Association (JAMA) of 214 patients with COVID-2019 from Wuhan, China, neurologic symptoms were seen more frequently than anticipated. About 36.4% of patients had some neurological symptoms, and they were more common in patients with severe infection (45.5%). The severe infection was described according to their respiratory status [7]. They also had more inflammation as indicated by a higher white blood cell (WBC) count, increased C-reactive protein (CRP), and d-dimer levels. They were also more likely to have multi-organ involvement like acute kidney injury, thrombocytopenia, and some soft of cardiomyopathy.

Vestibular neuritis, also known as vestibular neurolabyrinthitis or acute peripheral vestibulopathy is usually a benign, self-limited condition that presents with vertigo, nausea, vomiting, and sometimes gait impairment. It is a viral or post-viral inflammatory disorder affecting the vestibular portion of the eighth cranial nerve [8]. The clinical findings in these patients are consistent with an acute vestibular imbalance, just like how our patient has presented to the hospital. Our patient presented with significant vestibular symptoms and did not have any elevation in the inflammatory markers or other laboratory parameters. This finding correlates with the case series report where the investigators found no major abnormalities in the laboratory findings in those presenting with nervous system involvement [7]. Based on the findings and clinical course of the patient, we presume that our patient had COVID-19-induced vestibular neuritis.

The principal differential diagnostic concern we had was to exclude an acute vascular event in the central nervous system affecting the cerebellum and/or brainstem [9]. Our patient did not have any other neurologic signs and symptoms (dysarthria, dysphagia, weakness, sensory loss, or facial droop) to explain at acute CVA. As this differential diagnosis represents a potentially immediately life-threatening condition, it is important to consider this possibility in every patient who presents with acute and intractable vertigo [10]. Neuroimaging with CT scan and magnetic resonance imaging (MRI) excluded the diagnoses in our case, though the imaging could carry unto 12% false-negative results in acute ischemia [11]. A diagnosis of vestibular neuritis is largely based on the clinical presentation of an acute, sustained vestibular syndrome with examination features consistent with a peripheral lesion as discussed above. There are typically no specific diagnostic tests [12].

Neuroimaging is indicated to rule out alternative diagnoses if the examination is not entirely consistent with a cranial nerve lesion if there are prominent risk factors for stroke, if there are focal neurologic signs or symptoms, or if there is a new headache accompanying vertigo [10]. In our case, the patient was slightly hypertensive in the earlier stages of presentation, probably due to the intractable symptoms. This led to a suspected stroke and was ruled out based on the imaging. Though nystagmus increases the sensitivity of the diagnosis of acute vestibular neuritis, our patient did not exhibit nystagmus, which further added to the complexity of identifying the diagnosis.

Irrespective of the cause, potential treatments for vestibular neuritis from any etiology is symptomatic treatment and vestibular rehabilitation if needed. Corticosteroids could be used in severe cases [13].

Most of the literature suggests symptomatic treatments to reduce vertigo, nausea, and vomiting in the first few days of vestibular neuritis [10]. These include anti-emetics, antihistaminic, anticholinergics, and benzodiazepines, and an intravenous route is generally done in the initial stages, as the oral intake of these patients is poor. Vestibular exercises have been shown to be efficacious in improving symptoms and functioning as measured by a variety of symptoms and examination-based scores in patients with a unilateral peripheral vestibular injury [14].

Administration of steroids in the early course of COVID-19 was still controversial during her hospital stay as per the available literature during that time frame [15]. The institutional protocol and the available literature recommended the use of corticosteroids at least five to seven days after the onset of symptoms for an acute COVID-19 infection. It was indicated for acute COIVD-19 patients when there is no response to the commonly used and recommended treatment with hydroxychloroquine and azithromycin [5,14]. The management of our patient followed the traditional course of symptomatic medicines, corticosteroids, and vestibular rehabilitation. There was no indication to treat her with hydroxychloroquine or azithromycin, as she did not have any fever or respiratory symptoms and all her inflammatory marker numbers were normal [14]. However, the lack of literature during the early spike of COVID-19 cases in our facility encouraged us to use the azithromycin and hydroxychloroquine.

## Conclusions

This is a common cause of acute vestibular neuritis, but the patient presented to the hospital in the middle of a pandemic with a novel and deadly COVID-19 viral infection. This case suggests the importance of having an index of suspicion for a COVID-19 infection in patients presenting with upper respiratory and vestibular symptoms and adds valuable information to the limited literature on COVID-19 presentation and management.
